# The role of leadership identity in business and public sectors: a content analysis approach

**DOI:** 10.3389/fpsyg.2024.1492443

**Published:** 2025-06-20

**Authors:** Sevinc Engin, Fevzi Kasap

**Affiliations:** ^1^Department of Media and Communication Studies, Faculty of Communication, Near East University, Nicosia, Cyprus; ^2^Department of Radio, Television Cinema, Faculty of Communication, Near East University, Nicosia, Cyprus

**Keywords:** leadership identity, professional roles, identity construction, leadership conceptualization, qualitative analysis, business leaders

## Abstract

**Background:**

Leadership is a multifaceted concept shaped by professional, cultural, and organizational contexts. This study examines how business leaders conceptualize leadership and its influence on the construction of leadership identities. By focusing on sector-specific perspectives, the research highlights the varying definitions and roles of leadership across different professional environments.

**Method:**

A qualitative approach was employed to analyze leadership narratives from 71 participants, including CEOs, entrepreneurs, media professionals, politicians, and bureaucrats. Data were collected through in-depth interviews, and content analysis was used to identify themes and patterns in how leaders perceive and define their roles.

**Results:**

The findings reveal sectoral variations in leadership conceptualizations. Business leaders and entrepreneurs emphasize innovation, success, and strategic thinking. Journalists and media professionals prioritize influence and communication, while bureaucrats and politicians focus on ethics, public service, and societal values. These distinct perspectives demonstrate how leadership identities are shaped by the normative pressures and expectations of different professional contexts.

**Conclusion:**

The study underscores the contextual nature of leadership perceptions and their role in shaping leadership identity. It provides valuable insights into how leadership is constructed and understood across sectors, contributing to leadership theory and practice. Future research should expand the scope to include a wider range of professional groups and conduct comparative analyses to deepen understanding of the factors influencing leadership development.

## 1 Introduction

Leadership has been an essential part of human societies and continues to attract attention. Despite extensive research since the early 20th century, leadership still lacks a clear and unified definition, as leadership develops and is shaped by cultural, social, and organizational factors, making it difficult to define it with certainty. The well-known definition of Yukl emphasizes the role of leadership in influencing tasks and creating group cohesion to achieve goals. It also involves shaping culture and group identity (Yukl, 1989). While leadership is seen as setting goals and creating a shared vision, it is also associated with governance, team building, communication, and motivation (Collins, 2001; Zheng et al., 2021; Alga, 2017). Leadership and management have always been intertwined; leadership is often associated with influencing and inspiring followers, while management focuses on administrative functions (Nienaber, 2010).

Leadership often influences group actions to achieve shared goals (Bilgilier and Cetin, 2020). Leaders gain power through knowledge, personal traits, and authority; they can use rewards and punishment to maintain this power (Kilinc, 2012). Turkish leaders emphasize trust and influence in their leadership practices, especially in educational and political contexts (Alga, 2017). The political dimension of leadership is closely linked to the ability of political leaders to direct and influence society as they shape national and international policies through their vision and authority (Findikci, 2012). Political leadership plays an essential role in shaping societal outcomes, and political leaders guide their societies through their ideologies and influence their followers (Yolcu, 2019). Unlike social leadership, political leadership is based on political ideologies and often reflects the goals of a political party or movement. Political leaders represent their communities and use their power to influence public opinion, politics, and governance (Findikci, 2012).

With the rise of mass media, the image of leadership rather than political ideology has come to the fore. Political leaders often work with professionals to build their image and attract voters (Can and Gullupunar, 2020). The media is critical in this process, becoming indispensable for the press (Balci et al., 2020). Personal characteristics of political leaders, such as body language, speech patterns, and behaviors, are frequently scrutinized by voters; thus, political communication plays a crucial role in shaping voter preferences (Polat and Kulter, 2008).

In recent years, leadership has come to the fore in the field of education as well as in the field of politics. Education leaders are tasked with creating a vision for schools, fostering collaboration, and guiding institutions through times of change. Leadership in education entails balancing contrasting skills, such as adaptability and determination, to create an environment conducive to learning and innovation (Ulferts, 2021). The Multidimensional Leadership model sees leadership in education as a vital factor in managing the transformative changes needed in the 21st century (Gunbayi, 2019). Multidimensional leadership emphasizes the need for educational leaders to adjust their behavior to the situation and apply the most appropriate leadership style for each context. Leaders with this capacity can overcome complex challenges and promote a positive organizational culture (Kaplan and Kaiser, 2003). In today’s digital age, leaders in social media are now role models for students, creating an environment where digital information and social interaction play crucial roles in learning (Bates, 2019).

## 2 Literature review and research hypotheses

### 2.1 Theoretical framework

Our study’s academic and practical value will be its contributions to Leadership theory. In this context, linking our study more strongly with existing leadership models requires a broader approach to leadership. Yukl’s (1989) model, which defines leadership as an effective process to ensure group cohesion and achievement of goals, is essential in the leadership literature.

Yukl’s model emphasizes how leaders play a guiding role in achieving common goals by increasing cohesion within the group. On the other hand, this study focuses on the leadership perceptions of Turkish leaders and examines the roles of leaders in the direction and goal-setting processes in detail. By comparison with Yukl’s theory, these findings of the study may offer new perspectives on how cultural and contextual differences play a role in leadership practices.

Turkish leadership perceptions focus primarily on leaders building teams, setting visions, and strengthening their interactions within the group. In this respect, the study’s findings can detail how leaders support group cohesion in their decision-making processes and how this cohesion impacts achieving goals. For example, the strategies used by the leaders in the study to increase the motivation of the individuals in the group or the processes of creating a shared vision can make an essential contribution to leadership theory. This contribution emphasizes that leadership should be treated as an individual ability and a social process.

In addition, the post-industrial leadership model of Ziegler and DeGrosky (2008), which considers leadership as a collective process, provides a suitable point of comparison for the study’s findings. This model defines leadership through interactions and collective processes within a group rather than an individual role. The study’s findings on constructing leadership identity may reveal how personal and collective identities are intertwined. For example, data on how leaders evaluate not only their abilities and experiences but also group dynamics and the contributions of other team members may support the theory of Ziegler and DeGrosky (2008). In this context, the data obtained in the context of Turkish leadership adds a cultural dimension to the post-industrial leadership model and offers the opportunity to address the theory more comprehensively.

Cultural and contextual contributions also provide an essential opportunity to enrich leadership theory. While most of the leadership literature is based on Western-centered studies, the findings of this study are specific to the Turkish context and add a cultural dimension to the concept of leadership. The importance of trust, effective communication, and motivation in Turkish leadership perceptions can be associated with cultural factors. Especially in the context of political leadership, the roles of leaders, such as directing society, forming public opinion, and directing national policies, can be added to theoretical frameworks (Findikci, 2012; Yolcu, 2019). These contextual contributions can enable leadership theory to gain a broader meaning at a global level.

The study’s findings will allow the development of new conceptual models for leadership identity construction. Leadership identity can be defined within the framework of individual characteristics (self-confidence, motivation), social interactions (teamwork, communication), and contextual factors (culture, politics). Within the framework of identity arrangements proposed by Alvesson and Willmott (2002), a model can be developed on how leaders shape their identities according to continuous feedback and experiences. This model can allow for a better understanding of leadership identity’s dynamic and transformation-oriented nature.

Clarifying existing gaps in leadership theory is also essential in broadening the contribution. The difference between leadership and management, which is frequently discussed in the literature, can be addressed more clearly in light of the findings of this study. The distinction between leadership and management can be better explained by emphasizing the unique aspects of leadership, such as inspiring, visioning, and motivating (Nienaber, 2010). In addition, areas such as educational leadership and leadership in the digital age offer ideal areas to broaden the scope of theoretical contribution. The importance of transformation management and collaboration processes in leadership in education can be among the key elements that can be added to leadership theory (Gunbayi, 2019; Bates, 2019).

Finally, these theoretical contributions can provide essential clues to leadership practices. In the context of educational leadership, how leaders can implement change management and collaboration strategies can be discussed. In political leadership, how leaders manage their decision-making processes in the social context and their effects on public opinion can be added to the theoretical framework. These contributions will enhance not only the theoretical but also the practical impact of the study.

### 2.2 Study aim

Leadership is a dynamic, multifaceted structure encompassing influence, identity, and adaptability. Whether in political, educational, or organizational settings, leaders must constantly shape their identities and influence their followers to achieve shared goals. Current research continues to examine the evolving nature of leadership, particularly about trust, motivation, and organizational change. In the process, leaders continually reshape their identities based on feedback experiences and ongoing interactions between their followers (Alvesson and Willmott, 2002). Leaders are constantly shaping and revising their identities based on feedback from others and their self-perceptions. This study explores how leaders in various sectors perceive leadership, including business leaders, CEOs, company founders, senior executives, politicians, bureaucrats, and journalists. The goal is to uncover how these leaders describe themselves and how they perceive others they consider leaders. By analyzing these self-descriptions, the study seeks to understand how leadership identity is constructed and how different leaders develop their sense of leadership identity within their professional and social contexts. Prominent figures in Turkey-from business to politics-provide a unique perspective on leadership identity construction. These leaders navigate their roles while subjecting various social, cultural, and organizational pressures. The study delves into the conceptualizations of leadership that emerge from these different professional backgrounds and seeks to understand how these conceptualizations contribute to forming a leader’s identity. This study’s nuanced understanding of leadership will contribute to educational practices and leadership development in various sectors.

## 3 Data sources and research methods

This study explores how leaders construct and develop their self-identity by emphasizing similarities within their groups and differentiating themselves from others (Alvesson and Willmott, 2002; Bryman, 2004). Given the study’s focus on personal narratives and perceptions, a qualitative research method was deemed the most appropriate to achieve the research objectives. Leaders were asked to reflect on their understanding of leadership and share their experiences shaping their leadership identity. The data collected from these reflections were subjected to content analysis, a qualitative technique to identify patterns, themes, and insights from the narratives.

### 3.1 Data collection

In this research, a careful sampling strategy was adopted to examine how leaders construct their leadership identities and the factors that shape these identities. Participants consist of individuals who are considered leaders in their fields. The sample included 71 respondents grouped into three main groups: 29 company founders, CEOs, and senior executives; 20 journalists; and 22 politicians and bureaucrats. These groups were chosen to capture the perspectives of individuals who experience leadership roles in different contexts. The professional diversity of the participants provided a large data set for understanding the differences and similarities in the understanding of leadership.

In the selection of participants, individuals who are actively engaged in leadership roles were selected. The main criterion for determining the participants as leaders is that they are well-known and influential individuals in their professions. Company founders and CEOs represent strategic decision-making processes in corporate leadership. Journalists reflect the understanding of leadership from a different perspective with their impact on the media and public perception. Participants in politics and bureaucracy represent leadership roles in societal decision-making. Thus, this diversity has provided the opportunity to examine how leadership identity is perceived in different contexts.

Semi-structured interviews with the participants aimed to reveal in detail their understanding of leadership and their personal experiences behind this understanding. The fact that the interviews were conducted in Turkish allowed the participants to communicate comfortably and to preserve the cultural and contextual nuances in their expressions. This approach has led to a comprehensive consideration of individual and professional perspectives on the concept of leadership. This strategic selection process used in the research has created a solid basis for examining the perception of leadership in different professional fields and has increased the richness of qualitative data.

The translation process used in this study has been carefully planned and implemented to maintain the integrity of the meaning and context while translating the Turkish interviews into English.

All interviews were conducted in Turkish, which is the native language of the participants, so that the details, cultural meanings, and subtleties of the participants’ expressions were better captured. During the decipherment phase of the interviews, a careful word-by-word transcription process was applied to reflect what the interviewees said accurately. Explicit coding was carried out on the transcribed Turkish texts, and care was taken to preserve the context during the coding process. In this process, a context-oriented method was adopted instead of literal translation to accurately convey the cultural and linguistic contexts of the expressions used in English (Graneheim and Lundman, 2004). During coding, the texts were subjected to a multi-faceted examination so that the participants’ views on leadership characteristics, leadership perception, and the role of leaders did not lose their original meaning.

The researchers used a combination of various quality control methods to ensure the accuracy of the translation process. In the first stage, the translations were reviewed in detail by multiple researchers, and at this stage, cross-validation was performed to prevent possible translation errors or loss of meaning. In case of any contradictions or discrepancies identified during the translation process, the researchers have reached a consensus on the most appropriate translation by discussing the context in detail. In addition, the reverse translation method was applied to evaluate the accuracy of the translations. Some texts were re-translated from English into Turkish, and these new texts were compared with the original Turkish texts, and their consistency was evaluated. This reverse translation method has been used as an essential tool in improving the accuracy of the translation process (Krippendorff, 2004). These comprehensive translation evaluation processes, especially on themes such as leadership characteristics, context, and leadership actions, prevented the loss of meaning of the data. Thus, it was ensured that the expressions used during coding and theming were transferred correctly without breaking away from the context and preserving the integrity of the meaning.

### 3.2 Interview process

The semi-structured interviews involved open-ended questions encouraging participants to naturally discuss their experiences and perspectives. These questions were designed based on prior qualitative research on leadership (Alvesson and Sveningsson, 2003; Andersen, 2000; Nienaber, 2010), ensuring relevance and depth in responses. Table 1 outlines the four core questions that guided the interviews, though the exact wording and sequence of questions varied according to the course of each conversation. This flexibility allowed for a more organic exploration of each participant’s leadership journey and the factors that shaped their self-identity as leaders.

**Table 1:** Interview questions.

Interview Questions
How do you define a leader?
What makes a leader?
What are the qualities of a leader?
What contributes to individuals becoming leaders?
How do you think leaders emerge?
What qualities define a leader?

The study’s open-ended nature fostered in-depth discussions. It enabled participants to provide detailed insights into how they perceive leadership, their challenges, and how they developed their leadership skills. This method ensured that the data captured was rich, contextual, and highly reflective of each participant’s unique experience.

### 3.3 Data analysis

After the data collection phase, the content analysis methodology was used to identify themes and patterns in the participants’ narratives. Content analysis is a systematic coding and categorization approach used in qualitative research to interpret textual data. The transcribed interviews were thoroughly examined to uncover recurring themes related to leadership identity construction, influence, and the role of external and internal factors in shaping leadership behaviors.

The research team followed a thematic analysis approach, which involved reading and re-reading the transcripts to identify recurring patterns and essential themes. These themes were then grouped into broader categories to draw meaningful conclusions. Multiple research team members cross-checked the translated themes and categories to ensure accuracy and reliability. Qualitative analysis conducted by individuals can yield more meaningful results than software programs (Weitzman and Miles, 1995). However, qualitative research software is essential in organizing and retrieving data. In the study, coding and data management processes were carried out on Turkish texts using MAXQDA software, which provided a comprehensive platform for organizing, coding, and analyzing qualitative data. The software’s Turkish interface facilitated seamless coding, allowing researchers to work directly in the source language without losing cultural or contextual nuances. MAXQDA’s visual tools were employed to represent codes and themes graphically, enabling researchers to map relationships and overlaps between codes through visual diagrams and hierarchical frameworks.

To ensure accuracy, themes, and codes were first verified within the Turkish texts using MAXQDA’s retrieval functions, which allowed researchers to review specific coded segments in their original context. These verified codes were then carefully translated into English. The translation process involved detailed cross-referencing between Turkish and English texts to preserve meaning and context. MAXQDA’s side-by-side comparison feature was instrumental in this step, as it allowed researchers to juxtapose the Turkish codes with their English equivalents, ensuring consistency.

Additionally, MAXQDA’s memo and annotation features enhanced the collaborative coding process, which enabled researchers to document their discussions and rationale for specific coding decisions directly within the software. These detailed records helped resolve discrepancies and refine the coding scheme. The systematic application of MAXQDA’s tools throughout the coding and translation phases minimized the potential loss of meaning due to language differences and significantly enhanced the reliability of the research findings (Weitzman and Miles, 1995; Strauss, 1987).

Another critical stage of the research, inter-rater reliability, was used to measure consistency between researchers. During the coding process, each researcher independently determined the themes and codes, and then the level of compatibility between these codes was determined. The mediator reliability rate calculated with the Krippendorff Alpha coefficient used in the study was 85%. This high rate shows that the agreement among the researchers is quite strong, and reliable results are obtained in the coding process. Researchers have reached a consensus in disputed cases by discussing the relevant statements in their context (Graneheim and Lundman, 2004; Weitzman and Miles, 1995). These processes increased the overall validity of the research and allowed meaningful results to be obtained regarding the construction of leadership identity.

In this study, member-checking procedures were not applied, and the reason for not using this method is related to the characteristics of the research design and data collection process. The data collected in the study include the participants’ narratives and perceptions of leadership. Therefore, the purpose of the data was to explore the participants’ leadership experiences and perceptions as they were and to create thematic patterns from these narratives. Since the focus is more on these themes and categories, the focus is on the integrity of the data rather than checking the accuracy of individual opinions. In addition, the fact that the participants consisted of individuals with busy schedules, such as business leaders, journalists, and politicians, made it impractical to conduct participant verification due to logistical challenges.

The study applied alternative quality assurance methods instead of participant verification. First, multiple researchers independently coded the data, and discussions among the researchers reconciled the resulting differences between these codes. This collaborative coding process has contributed to ensuring objectivity in the interpretation of the data. In addition, mediator reliability calculations were made to evaluate the consistency of the coding process, and in this process, a Krippendorff Alpha coefficient of 85% was reached. This high rate indicated a strong concordance among researchers and a reliable analysis process. The reverse translation method was applied in the translation process, the texts retranslated from English to Turkish were compared with the original Turkish texts, and the compatibility was evaluated.

Finally, the consistency of the themes obtained in context and meaning was ensured through in-team discussions and rich descriptions in the research. The research team reviewed the themes through regular meetings and conducted an intensive analysis process to prevent loss of meaning during translation. Other robust verification methods have compensated for the lack of participant verification in these processes. This careful approach to coding and translation processes has minimized the loss of meaning from language differences, allowing the study to provide reliable and valid results.

## 4 Results and discussion

This study analyzed data using content analysis (Table 2). Content analysis examines the presence and relationships of specific words and phrases in communication to draw inferences about the message (Nienaber, 2010). The analysis process involved identifying text blocks, defining the unit of analysis, conducting open coding, creating a coding scheme, and identifying patterns. Data was collected through face-to-face interviews with 71 leaders, and the transcribed interviews served as the research data. Words and phrases relevant to the research questions were highlighted and coded (Krippendorff, 2004). Researchers conducted the coding process individually and then collaborated to reach a consensus on the coding scheme after the open coding phase. Following this, axial coding was conducted, involving coding around specific categories. Any discrepancies were thoroughly discussed to ensure the reliability of the analysis, and an agreement was reached on the coding of each theme (Graneheim and Lundman, 2004).

**Table 2:** Coding scheme analysis and commentary.

Coding category	Category description
Actor	*Individuals as actors*: This includes the names of individuals, characters, and similar concepts (e.g., person, individual, manager, leader, etc.) or personalities (e.g., Ataturk, Ozal, Demirel, etc.) *Groups of people as actors*: This covers family, women, community, society, and people in general *Institutions and entities as actors*: This encompasses government, companies, and similar concepts (e.g., institution, entity, company, etc.) or specific entities (e.g., Turkish Industry and Business Association (TUSIAD), etc.)
Leader qualities	Leadership qualities include knowledge, honesty, courage, self-discipline, and similar
Leader activities	Leaders execute leader activities, and the processes they engage in include goal-setting, problem-solving, decision-making, and similar activities
Achievements	Success, power, efficiency, change
Context	*Context as a region:* Region, countries, locations, places, and similar as a concept (such as city, country, etc.) or location (such as Republic of Turkey, Istanbul, Jerusalem, etc.) *Context as an idea or event:* Political context, societal context, environment, Internet, industry, etc.
Circumstances	Democracy, economic condition, and similar concepts as broader and undefined concepts (such as condition, uncertainty, etc.) or specifically defined concepts (such as social conditions, financial conditions, historical background, etc.)
Law	These concepts emphasize legal systems such as law, rights, constitution, justice, etc.
Politics	Concepts that emphasize politics include candidates, parliament, and similar
Religious emphasis	Concepts that emphasize religion include Islam, the Koran, the prophet, religion, and religion
Media	Concepts that emphasize media include journalism, newspaper, publication, and similar

The coding scheme in Table 2 organizes and categorizes leadership identity and activities, offering a systematic approach to analyzing qualitative data from interviews with leaders. Categories in the Coding Scheme are:

*Actor:* This category identifies the key figures involved in leadership as mentioned by participants, including individuals, groups, and institutions. Individuals as actors include notable historical and contemporary figures, such as political and business leaders like Atatürk, Özal, and Demirel. Groups of people, such as family, women, and the community, reflect a collective view of leadership. Additionally, institutions and organizations, such as the government and business entities like the Turkish Industry and Business Association (TUSIAD), are seen as significant actors in leadership. This broader perspective on leadership suggests that leadership identity is shaped by individual leaders, groups, and institutions, reflecting the historical and cultural contexts in which leadership occurs.

*Leader qualities:* This category emphasizes the traits commonly attributed to leaders, such as knowledge, honesty, courage, and self-discipline. The qualities most often associated with leadership emphasize ethical integrity and competence. Traits like honesty and courage suggest that leaders are expected to embody moral authority, while self-discipline and knowledge highlight the importance of strategic and practical skills in effective leadership.

*Leader activities:* Leadership encompasses the actions undertaken by leaders, including goal-setting, problem-solving, and decision-making. Leadership is inherently action-oriented. These activities emphasize that leadership is not solely about possessing certain traits but also about executing tasks that mold organizations and influence others.

*Achievements:* This category pertains to the results that leaders are expected to achieve, such as success, power, efficiency, and change. The analysis shows that leadership is linked to attaining specific, concrete outcomes. This is consistent with the belief that leaders are accountable for advancing progress, whether innovating in business or effecting change in political settings.

*Context:* Leadership does not exist in a vacuum; it is influenced by external factors such as geographical locations, political contexts, and societal environments. The environment in which leadership is practiced significantly shapes leadership identity. Leaders face different demands based on their regions or societal settings, and these factors must be considered when evaluating leadership effectiveness.

*Circumstances:* This category encompasses overarching societal factors such as democracy, economic conditions, and social circumstances. Leadership is influenced by systemic forces that can either empower or limit a leader’s actions. For example, leaders must adeptly navigate the obstacles and possibilities of democratic systems or economic changes.

*Law:* Leadership is frequently examined within the legal framework, placing significant emphasis on rights, constitution, and justice. The legal aspect of leadership underscores the significance of justice and fairness in carrying out leadership responsibilities. This indicates that ethical governance and compliance with laws are essential to leadership in political and bureaucratic settings.

*Politics:* In political arenas, leadership is intricately linked with governance and the electoral process. Political leaders must adeptly navigate intricate legislative and electoral systems pivotal to their leadership roles and responsibilities.

*Religious emphasis:* In specific environments, religion influences leadership through concepts such as Islam, the Koran, and the Prophet. Religious principles can shape leadership identity, particularly in societies where religion holds significant importance. Leaders may be anticipated to uphold religious values and embody spiritual authority alongside their leadership responsibilities.

*Media:* This section delves into the influence of journalism, publications, and media outlets on shaping perceptions of leadership. The media plays a vital role in shaping leaders’ identities. Their portrayal in the media often forms the basis for judgment, and media platforms are essential for leaders to communicate and bolster their public image.

Table 2 presents a structured approach to analyzing leadership across various contexts, demonstrating its complexity as a social construct influenced by social, political, legal, and cultural factors. The coding scheme highlights the importance of integrating personal traits with external influences, as effective leadership requires understanding both individual attributes and the broader context, aligning with views from scholars like Yukl (2022) and Northouse (2021). It also emphasizes the role of media in shaping leadership identity, supporting the argument of Fairhurst and Connaughton (2014) about leaders’ reliance on media. Additionally, it underscores the significance of societal structures, such as religious, political, and legal frameworks, in influencing leadership. Thus, the table not only organizes the data but also unveils the multifaceted nature of leadership, shaped by both individual qualities and external factors.

Table 3 presents the distinctive themes identified from interviews with politicians, detailing their frequencies, percentages, and weighted percentages relative to all themes across categories. This quantitative breakdown provides a clearer understanding of the prevalence and significance of each theme within the broader context of leadership discussions. Weighted percentages are calculated to better understand the relative significance of each category or theme within the overall dataset, considering its impact or importance relative to other themes. This method accounts for data distribution across multiple categories and normalizes the values to make them comparable. Weighted percentages provide several key advantages in data analysis. They enable a holistic understanding by comparing the prominence of themes across categories, not just within individual groups, offering a broader perspective. Additionally, they normalize data, facilitating cross-category comparisons and revealing overarching patterns more effectively. By incorporating the entire dataset, weighted percentages also highlight the relative importance of each theme in the overall context, ensuring a clear and accurate representation of findings.

**Table 3:** Distinctive themes derived from the interviews with politicians and bureaucrats.

Actor	*F*	%	Weighted %		*F*	%	Weighted %
Community	58	5.53	2.47	Society	114	10.87	4.86
Person	146	13.92	6.22	Citizen	18	1.72	0.77
Public	5	0.48	0.21	Administrator	19	1.81	0.81
Mustafa Kemal Atatürk	18	1.72	0.77	Family	51	4.86	2.17
Leader	235	22.40	10.01	AKP	28	2.67	1.19
Menderes	6	0.57	0.26	Ecevit	10	0.95	0.43
Manager	8	0.76	0.34	Allah	70	6.67	2.98
Omer	8	0.76	0.34	Anavatan (political party)	12	1.14	0.51
Ottoman	31	2.96	1.32	Bureaucrat	6	0.57	0.26
Ozal	12	1.14	0.51	Employee	7	0.67	0.30
Sultan	25	2.38	1.07	Municipality	65	6.20	2.77
Pasha	12	1.14	0.51	Army	14	1.33	0.60
Owner	25	2.38	1.07	Prime Minister	12	1.14	0.51
Yavuz Sultan Selim	8	0.76	0.34	President	10	0.95	0.43
Demirel	16	1.53	0.68	**Total**	**1,049**	**100.00**	**44.70**
**Politics**	***F***	**%**	**Weighted %**	**Context**	***F***	**%**	**Weighted %**
Candidate	13	2.93	0.55	United States of America	16	4.92	0.68
Political party	100	22.52	4.26	Anatolia	9	2.77	0.38
Coup	8	1.80	0.34	City	5	1.54	0.21
Party in power	60	13.51	2.56	Republic of Turkey	74	22.77	3.15
Cabinet	12	2.70	0.51	Country	56	17.23	2.39
Ideology	6	1.35	0.26	Government	40	12.31	1.70
Political position	5	1.13	0.21	World	51	15.69	2.17
Coalition	24	5.41	1.02	Jerusalem	12	3.69	0.51
Government agency	5	1.13	0.21	Cyprus	8	2.46	0.34
Parliament	10	2.25	0.43	Egypt	6	1.85	0.26
Governmental policy	5	1.13	0.21	Medina	10	3.08	0.43
Vote	12	2.70	0.51	Mecca	8	2.46	0.34
Elections	67	15.09	2.85	Palestine	8	2.46	0.34
System	66	14.86	2.81	Israel	6	1.85	0.26
Politics	27	6.08	1.15	Istanbul	16	4.92	0.68
Democrats	15	3.38	0.64	**Total**	**325**	**100.00**	**13.85**
Seat	9	2.03	0.38				
**Total**	**444**	**100.00**	**18.92**				
**Leadership activities**	***F***	**%**	**Weighted %**	**Leadership qualities**	***F***	**%**	**Weighted %**
Offering solutions	13	6.07	0.55	Knowledgeable	9	8.57	0.38
Problem-solving	7	3.27	0.30	Courageous	5	4.76	0.21
Building trust	7	3.27	0.30	Attentive	15	14.29	0.64
Governance	19	8.88	0.81	Honest	6	5.71	0.26
Articulation	12	5.61	0.51	Clever	33	31.43	1.41
Decision making	9	4.21	0.38	Personality	5	4.76	0.21
Control	8	3.74	0.34	Sophisticated	7	6.67	0.30
Crisis management	6	2.80	0.26	Intelligent	25	23.81	1.07
Team building	7	3.27	0.30	**Total**	**105**	**100.00**	**4.47**
Implementation	6	2.80	0.26				
Fulfilling expectations	8	3.74	0.34				
Administration	61	28.50	2.60				
Resolution	51	23.83	2.17				
**Total**	**214**	**100.00**	**9.12**				
**Circumstances**	***F***	**%**	**Weighted %**	**Religious emphasis**	***F***	**%**	**Weighted %**
Democracy	40	34.19	1.70	Islam	24	26.37	1.02
Economic conditions	9	7.69	0.38	Koran	7	7.69	0.30
Social circumstances	33	28.21	1.41	Prophet	18	19.78	0.77
Historical background	18	15.38	0.77	Religion	5	5.49	0.21
Conditions	7	5.98	0.30	Worship	5	5.49	0.21
Uncertainty	10	8.55	0.43	Muslim	32	35.16	1.36
**Total**	**117**	**100.00**	**4.99**	**Total**	**91**	**100.00**	**3.88**
**Achievements**	***F***	**%**	**Weighted %**	**Law**	***F***	**%**	**Weighted %**
Success	38	32.48	1.62	Constitution	18	19.35	0.77
Value	14	11.97	0.60	Rights	17	18.28	0.72
Service	12	10.26	0.51	Legislation	20	21.51	0.85
Responsiveness	12	10.26	0.51	Justice	38	40.86	1.62
Efficiency	6	5.13	0.26	**Total**	**93**	**100.00**	**3.96**
CSR	6	5.13	0.26				
Power	29	24.79	1.24				
**Total**	**117**	**100.00**	**4.99**				

Table 3 analyzes key themes from interviews with politicians and bureaucrats, focusing on actors, political elements, leadership activities, qualities, and achievements. The analysis offers insight into factors shaping leadership identity in political and bureaucratic settings:

*Actors:* Prominent figures such as Mustafa Kemal Atatürk and political groups (e.g., AKP) are frequently mentioned. Leader (22.40%) highlights personalized leadership, with Atatürk as a lasting symbol of leadership. Community and society (16.4%) emphasizes leaders as representatives of public welfare. Leadership in political settings often revolves around significant individuals and their relationships with the broader society. Politicians and bureaucrats recognize leadership as a public service position, underlining the interdependence between leaders and their constituencies (Northouse, 2021; Celik and Yilmaz, 2021).

*Politics and political context:* Political terms reflect the role of institutions in shaping leadership. Political parties (22.52%) play a crucial role in shaping leadership identity through party affiliation. Elections (15.09%) are essential for validating leadership authority. Leadership is tied to institutional mechanisms like elections, which shape leaders’ public perception (Yukl, 2022; Genckaya and Kabasakal, 2024).

*Leadership activities:* These are crucial effectiveness indicators, particularly in governance, decision-making, and crisis management. Governance (8.88%) is a central leadership function requiring strategic action. Decision-making (4.21%) is crucial, as effective decisions shape political outcomes. Crisis management (2.80%) is critical, with leadership success often tied to navigating crises. Political leadership is characterized by a series of activities that go beyond symbolic representation; it demands action-oriented leadership with a focus on decision-making and crisis resolution (Akdemir, 2012; Chiwisa, 2024).

*Leadership qualities:* These are critical in politics, including knowledge, courage, and honesty. Knowledgeable (8.57%) leaders require a deep understanding of policy to be effective. Honesty (5.71%) is crucial, as integrity is critical to maintaining credibility. Courage (4.76%) is necessary for making high-stakes decisions. The emphasis on personal attributes such as knowledge, honesty, and courage underscores the multifaceted nature of political leadership. Influential leaders must balance intellectual capacity with moral integrity to navigate the complexities of governance (Wolff and Demir, 2019; Celik and Yilmaz, 2021; Sari, 2021).

*Circumstances:* Broader contexts, such as democracy and economic challenges, influence leadership. Democracy (34.19%) shapes leadership identity through democratic governance. Economic conditions (7.69%) affect leadership effectiveness, as leaders must manage economic challenges. Social circumstances (28.21%) influence leadership perceptions, especially during social unrest. Leaders are profoundly shaped by their environments. Democratic processes, financial performance, and social conditions provide the backdrop against which leadership is enacted and assessed (Gursoy, 2014; Kaplan, 2006; Colak and Keskin, 2022; Sahin, 2015; Cooper et al., 2024).

*Achievements:* Success, power, and responsiveness are crucial metrics for assessing political leadership. Success (32.48%) is often measured through policy outcomes or contributions to public service. Power (24.79%) is essential to leadership but must be exercised responsibly. Responsiveness (10.26%) to public concerns enhances the perception of effective leadership. Leadership achievements are not just about accumulating power but also involve tangible outcomes that benefit the public. Success in leadership is often determined by the ability to deliver meaningful change and maintain public trust (Cetin and Akdemir, 2023; Akar and Gulen, 2023; Dogan, 2015).

Analyzing themes from interviews with politicians and bureaucrats provides a comprehensive understanding of political leadership identity. Leadership is shaped by personal qualities, actions, and external circumstances, all of which contribute to the broader perception of a leader’s effectiveness. The findings highlight that leadership is about personal traits, navigating institutional structures, responding to societal challenges, and achieving tangible outcomes that resonate with the public.

The interviews identified five main theme categories: actors, leadership activities, qualities, achievements, and external contexts, as detailed in Table 4.

**Table 4:** Analysis and interpretation of themes from interviews with company founders, CEOs, and senior executives.

Actor	*F*	%	Weighted %	Leadership activities	*F*	%	Weighted %
Family	24	2.58	1.48	Team building	30	12.35	1.84
Mustafa Kemal Ataturk	36	3.88	2.21	Identifying opportunities	6	2.47	0.37
Individual	5	0.54	0.31	Goal setting	24	9.88	1.48
Employee	6	0.65	0.37	Ensuring communication	12	4.94	0.74
Person	112	12.06	6.88	Decision making	46	18.93	2.83
Women	31	3.34	1.91	Crisis management	6	2.47	0.37
Entity	21	2.26	1.29	Motivating	17	7.00	1.04
Institution	5	0.54	0.31	Being a role model	18	7.41	1.11
Leader	490	52.74	30.12	Planning	16	6.58	0.98
Partner	18	1.94	1.11	Problem-solving	11	4.53	0.68
Owner	13	1.40	0.80	Risk-taking	11	4.53	0.68
Company	36	3.88	2.21	Offering solutions	8	3.29	0.49
Society	34	3.66	2.09	Setting a corporate vision	13	5.35	0.80
TUSIAD	8	0.86	0.49	Investing	8	3.29	0.49
Manager	90	9.69	5.53	Governance	17	7.00	1.04
**Total**	**929**	**100.00**	**57.10**	**Total**	**243**	**100.00**	**14.94**
**Leader qualities**	***F***	**%**	**Weighted %**	**Achievements**	***F***	**%**	**Weighted %**
Just	7	4.22	0.43	Success	41	31.30	2.52
Knowledgeable	9	5.42	0.55	Value	12	9.16	0.74
Courageous	7	4.22	0.43	Change	15	11.45	0.92
Self-disciplined	5	3.01	0.31	Power	29	22.14	1.78
Emotionally intelligent	35	21.08	2.15	Trust	13	9.92	0.80
Educated	13	7.83	0.80	CSR	11	8.40	0.68
Empath	12	7.23	0.74	Service	5	3.82	0.31
Genetic qualities	5	3.01	0.31	Innovation	5	3.82	0.31
Characteristics	63	37.95	3.87	**Total**	**131**	**100.00**	**8.05**
Punctual	5	3.01	0.31				
Qualified	5	3.01	0.31				
**Total**	**166**	**100.00**	**10.20**				
**Context**	***F***	**%**	**Weighted %**				
World	39	24.68	2.40				
Environment	5	3.16	0.31				
Political context	22	13.92	1.35				
Industry	5	3.16	0.31				
Societal context	15	9.49	0.92				
Republic of Turkey	44	27.85	2.70				
Country	8	5.06	0.49				
Location	20	12.66	1.23				
**Total**	**158**	**100.00**	**9.71**				

Table 4 presents an analysis of leadership in corporate settings based on interviews with founders, CEOs, and managers, focusing on actors, leadership activities, attributes, achievements, and external contexts:

*Actors:* Corporate leadership emphasizes individual-centric approaches, where personal leadership is key. Leader (52.74%) is viewed as highly individualistic, with individual traits like charisma and vision being central to success. Personal characteristics (12.06%) such as determination and adaptability are vital to effective leadership. Family background (2.58%) plays a role in influencing leadership development and decision-making. Company (3.88%) ties the leader’s identity closely to the success of their organization. Business leadership is strongly individualized, but it also relies on personal backgrounds, family influence, and organizational contexts. Leaders do not operate in isolation; they are supported by various individual and institutional actors that contribute to their leadership identity (Schermerhorn et al., 2020; Demir, 2023; Yukl, 2022).

*Leadership activities:* Leadership activities highlight the operational processes that leaders engage in to influence organizational outcomes. Decision-making (18.93%) is a priority, as leaders focus on making timely and strategic decisions. Team building (12.35%) is essential, with collaborative leadership models playing a key role in modern organizations. Motivating (7.00%) is essential, as leaders inspire employees to achieve organizational goals. Crisis management (2.47%) is crucial, especially in managing crises within unstable environments. Corporate leadership is highly action-oriented, with decision-making being the most prominent activity. Leaders are evaluated on their ability to make informed decisions, build high-performing teams, and maintain employee motivation, especially in times of crisis (Goleman, 2020; Robbins and Judge, 2021; Yukl, 2022).

*Leadership qualities:* The qualities associated with effective leadership in the corporate world focus on a blend of cognitive and emotional competencies. Emotional intelligence (21.08%) is essential, as leaders must manage emotions to foster collaboration. Courage (4.22%) is required for high-stakes decision-making. Knowledge (5.42%) is necessary for leaders with industry expertise to guide strategy effectively. Empathy (7.23%) is increasingly valued in modern leadership, emphasizing inclusivity and understanding. Corporate leadership today is characterized not only by cognitive skills but also by emotional and relational competencies. Emotional intelligence and empathy suggest a move away from authoritarian leadership toward a more inclusive style that values interpersonal relationships and ethical responsibility (Goleman, 2020; Schermerhorn et al., 2020; Northouse, 2021).

*Achievements:* Corporate leadership is evaluated through tangible outcomes, such as Success (31.30%), which is primarily measured by profitability and market share, which remain key indicators. Value (9.16%) emphasizes leaders’ focus on generating long-term stakeholder value. Change (11.45%) highlights the critical importance of driving innovation. Corporate social responsibility (CSR) (8.40%) reflects the growing ethical expectations placed on leaders. While corporate leadership has traditionally been measured by financial success, modern leaders are also judged by their capacity to create long-term value and address corporate social responsibilities. This shift reflects broader societal demands for ethical business practices (Porter and Kramer, 2019; Goleman, 2020; Robbins and Judge, 2021).

*Context:* The context in which corporate leaders operate significantly shapes their strategies and decision-making processes. Global context (24.68%) requires adaptability, as corporate leaders must navigate multinational environments. National context (27.85%) shapes leadership strategies through policies and regulations. Political context (13.92%) influences corporate decisions, as leaders must consider political dynamics. Industry context (3.16%) requires leaders to address sector-specific risks and opportunities. Both global and local factors shape successful corporate leadership. Leaders must be adaptable to external pressures while aligning their strategies with industry-specific demands and national regulations (Porter and Kramer, 2019; Kaya, 2021; Yukl, 2022).

In summary, the themes derived from interviews with company founders, CEOs, and senior executives highlight that corporate leadership is multifaceted, requiring a combination of personal traits, strategic actions, and adaptability to complex environments. Leaders are expected to make critical decisions, build strong teams, and drive organizational success while balancing emotional intelligence, ethical responsibility, and global competitiveness. Corporate leadership requires a blend of personal traits, strategic actions, and adaptability. Leaders must make critical decisions, build strong teams, and drive success while balancing emotional intelligence and ethical responsibility. The modern corporate leader is measured by profitability and their ability to foster innovation, create long-term value, and contribute to societal well-being through CSR initiatives. This reflects a shift toward more human-centric, socially responsible leadership models.

Table 5 provides a thematic analysis based on interviews with journalists. It categorizes aspects of leadership such as actors, qualities, activities, context, achievements, media influence, and the circumstances surrounding leadership. The table also includes the frequency and weighted percentages of these themes to offer deeper insights into leadership in the media context.

**Table 5:** Analysis and interpretation of themes derived from interviews with journalists.

Actor	*F*	%	Weighted %	Leader qualities	*F*	%	Weighted %
Family	26	5.71	2.74	Knowledgeable	10	17.24	1.05
Mustafa Kemal Ataturk	12	2.64	1.26	Emotionally intelligent	9	15.52	0.95
Employee	5	1.10	0.53	Educated	9	15.52	0.95
Government	7	1.54	0.74	Attentive	5	8.62	0.53
Tayyip Erdogan	5	1.10	0.53	Punctual	9	15.52	0.95
Individual	43	9.45	4.53	Intelligent	16	27.59	1.69
Public	7	1.54	0.74	**Total**	**58**	**100.00**	**6.11**
Manager	39	8.57	4.11				
Institution	5	1.10	0.53				
Leader	259	56.92	27.29				
Society	47	10.33	4.95				
**Total**	**455**	**100.00**	**47.95**				
**Leadership activities**	***F***	**%**	**Weighted %**	**Context**	***F***	**%**	**Weighted %**
Team building	18	18.37	1.90	World	27	28.13	2.85
Goal setting	10	10.20	1.05	France	7	7.29	0.74
Perception management	6	6.12	0.63	India	6	6.25	0.63
Decision making	38	38.78	4.00	Internet	7	7.29	0.74
Governance	13	13.27	1.37	Republic of Turkey	43	44.79	4.53
Crisis management	8	8.16	0.84	Country	6	6.25	0.63
Problem-solving	5	5.10	053	**Total**	**96**	**100.00**	**10.12**
**Total**	**98**	**100.00**	**10.33**				
**Achievements**	***F***	**%**	**Weighted %**	**Media**	***F***	**%**	**Weighted %**
Success	41	41.84	4.32	Newspaper	20	23.81	2.11
Power	29	29.59	3.06	Journalist	12	14.29	1.26
Trust	7	7.14	0.74	News	5	5.95	0.53
Efficiency	5	5.10	0.53	Publication	8	9.52	0.84
CSR	5	5.10	0.53	Media	39	46.43	4.11
Change	11	11.22	1.16	**Total**	**84**	**100.00**	**8.85**
**Total**	**98**	**100.00**	**10.33**				
**Circumstances**	***F***	**%**	**Weighted %**				
Democracy	5	8.33	0.53				
Economic conditions	27	45.00	2.85				
Uncertainty	12	20.00	1.26				
Conditions	6	10.00	0.63				
Political conditions	10	16.67	1.05				
**Total**	**60**	**100.00**	**6.32**				

This analysis comprehensively explains how journalists perceive and interact with leadership. These themes are broken down into frequencies and percentages for a deeper understanding.

*Actors:* Journalists identify actors from individual figures to institutions and societal entities, emphasizing the interplay between personal leadership and broader societal structures. Leadership (56.92%) is primarily personified, with leaders being the central focus of media scrutiny and public narratives. Individual stories (9.45%) are emphasized by journalists, who use them to humanize broader societal issues. Public opinion (10.33%) plays a crucial role in leadership, as the media bridges leaders and society, shaping leadership perceptions. Personal background and familial support (5.71%) significantly influence how leadership is perceived personally and publicly. Media-government relations (1.54%) reflect the media’s complex coverage of political decisions and their societal impacts, with equal attention given to both the government and the public. Leadership in journalism is primarily personified, focusing on individual leaders while recognizing the importance of broader societal and institutional actors. Journalists serve as crucial intermediaries between leaders and society, facilitating a public understanding of leadership dynamics (Northouse, 2021; Robbins and Judge, 2021; Yukl, 2022).

*Leadership qualities:* Key leadership traits emphasized by journalists include: Intelligence (27.59%) is highly valued as it enables leaders to navigate complex environments effectively. Knowledge (17.24%) is essential for leaders to make informed decisions, as comprehensive understanding is key. Emotional intelligence (15.52%) plays a crucial role in leadership, as managing emotions is vital for leading effectively. Punctuality (15.52%) reflects professionalism and reliability, which are essential for meeting media expectations. Education (15.52%) is critical for understanding complex issues and earning respect, enhancing a leader’s credibility. Journalists prioritize intellectual and emotional capacities in leaders, highlighting the importance of balancing cognitive intelligence with emotional awareness. These traits are particularly crucial in high-stakes environments where leaders must navigate challenges and crises (Heifetz et al., 2009; Goleman, 2020; Yukl, 2022).

*Leadership activities:* Journalists assess leadership through key activities: Decision-making (38.78%) is the most essential leadership function, particularly in high-pressure situations where effective decisions are critical. Team building (18.37%) fosters organizational collaboration and efficiency. Governance (13.27%) requires leaders to ensure organizational goals are achieved through effective oversight and structure. Crisis management (8.16%) is a key leadership skill. Leaders are often judged by how well they handle crises, frequently attracting media attention. Perception management (6.12%) is crucial, as shaping public perception influences how leadership is portrayed in the media. Journalists emphasize decision-making and team-building as critical leadership activities. They also highlight governance and crisis management, recognizing that effective leadership is about navigating challenges while maintaining a positive public image (Robbins and Judge, 2021; Northouse, 2021; Yukl, 2022).

*Context:* The context influences leadership strategies and decision-making. World (28.13%) highlights the growing relevance of global challenges as leadership becomes more interconnected. The Republic of Turkey (44.79%) places local issues at the core of leadership discourse, focusing on how leaders address national challenges. Internet (7.29%) is transforming leadership dynamics and public engagement through digital platforms. France (7.29%) and India (6.25%) provide broader perspectives through comparisons with leadership in other countries. Leadership in journalism is analyzed within both global and local contexts, focusing on how leaders respond to international pressures and national challenges. The growing role of the internet highlights the shifting nature of media and its impact on leadership (Goleman, 2020; Robbins and Judge, 2021; Yukl, 2022).

*Achievements:* Leadership achievements are key to media evaluations. Success (41.84%) primarily focuses on tangible outcomes, such as success in politics or business. Power (29.59%) is crucial, as adequate power is key to driving change or maintaining control. Trust (7.14%) is vital for long-term leadership success, with public trust being a key factor. Corporate Social Responsibility (CSR) (5.10%) emphasizes the growing importance of ethical leadership and social responsibility in leadership evaluations. Journalists strongly emphasize success and power but also recognize the importance of trust and moral leadership. The inclusion of CSR in leadership evaluations reflects a growing expectation for leaders to prioritize social responsibility alongside traditional success metrics (Porter and Kramer, 2019; Northouse, 2021; Yukl, 2022).

*Circumstances:* External circumstances shape leadership challenges. Economic conditions (45.00%) are a significant influence on leadership decisions, shaping strategies and actions. Political conditions (16.67%) require leaders to navigate constantly changing political landscapes. Uncertainty (20.00%) tests leadership effectiveness, especially in managing crises and unpredictable situations. Democracy (8.33%) emphasizes how democratic pressures and public accountability shape leadership actions. Journalists recognize that external factors, such as economic and political conditions, greatly influence leadership. The ability to navigate uncertainty and respond to democratic pressures is a key indicator of effective leadership (Robbins and Judge, 2021; Northouse, 2021; Yukl, 2022).

In summary, the themes derived from interviews with journalists provide valuable insights into how the media perceives and evaluates leadership. Journalists emphasize key leadership qualities such as intelligence, emotional awareness, and knowledge while focusing on critical activities like decision-making, team-building, and crisis management. Achievements, particularly success and power, are central to leadership evaluations, but there is a growing emphasis on trust and corporate social responsibility.

Journalists recognize the complexity of leaders’ environments, considering economic, political, and societal factors. Their coverage reflects an understanding that leadership is about more than just achieving results-it also involves maintaining public trust and navigating uncertain circumstances. The increasing focus on CSR indicates that ethical leadership is becoming a more important criterion for evaluating leadership success.

**Figure 1:**
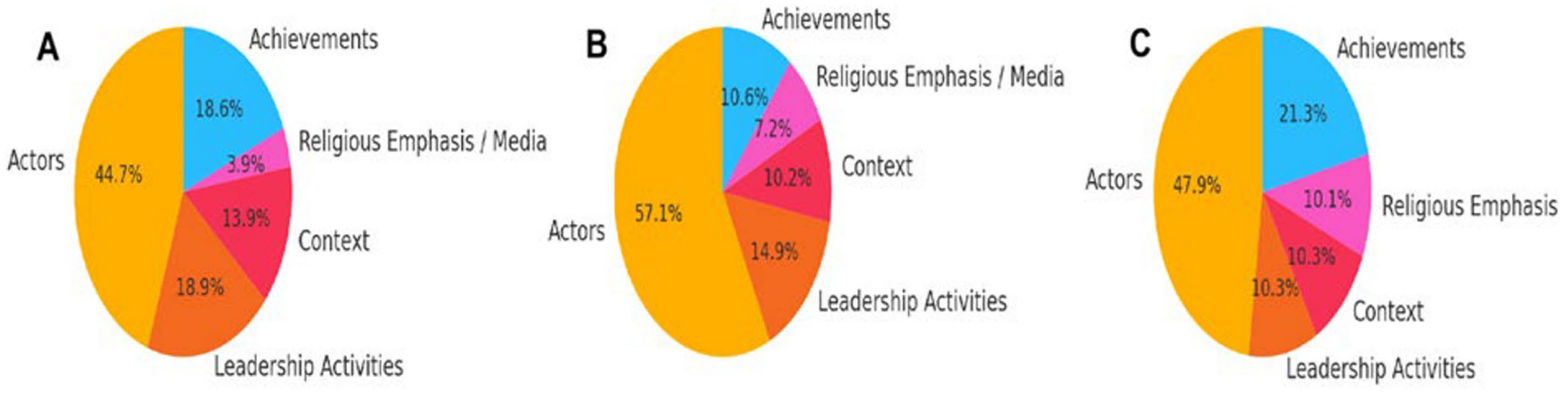
**(A)** Distribution of leadership themes for politicians. **(B)** Distribution of leadership themes for CEOs and senior executives. **(C)** Distribution of leadership themes for journalists.

Figure 1A visualizes the distribution of leadership themes derived from interviews with politicians. The data reveals that “actors” form the most significant category (44.70%), heavily emphasizing historical and contemporary political figures such as Mustafa Kemal Ataturk, Turgut Ozal, and Suleyman Demirel. This reflects politicians’ tendency to reference prominent leaders when discussing leadership. The second most frequently mentioned theme is “politics” (18.92%), underlining the association between leadership and concepts such as elections, democracy, and parliamentary functions. Interestingly, “religious emphasis” (3.88%) emerged as a unique theme, illustrating how politicians relate leadership to religious values and figures, a finding attributed to the conservative nature of the current government. Additionally, the theme of “context” (13.85%) indicates that politicians perceive leadership as highly dependent on the socio-political environment.

The dominance of historical figures in politicians’ conceptualization of leadership highlights the symbolic value these figures hold in Turkey’s political landscape. Furthermore, the association of leadership with political mechanisms such as elections and democratic processes underscores the role of governance and institutional structures in shaping political leadership. The emergence of religious themes suggests that political leadership is also informed by the intersection of religion and politics, which reflects the prevailing cultural and ideological frameworks.

Figure 1B illustrates the distribution of leadership themes conceptualized by company founders, CEOs, and senior executives. The most prominent theme is “actors” (57.10%), indicating that these corporate leaders view leadership primarily as individual figures. However, unlike politicians, they also emphasize organizational actors such as stakeholders, shareholders, and employees, reflecting a broader view of leadership within an organizational context. “Leadership activities” (14.94%) is the second most prominent theme, pointing to the importance of actions such as decision-making, team-building, and strategy formulation. The third theme is “leadership qualities” (10.20%), emphasizing personal traits like resilience, intelligence, and emotional intelligence as crucial for effective leadership.

The emphasis on individual actors in corporate leadership reflects the highly personalized nature of leadership in the business sector, where visionaries such as CEOs are seen as central to an organization’s success. However, the attention to organizational actors suggests a more complex view of leadership involving coordination among various stakeholders. The significant focus on leadership activities highlights the action-oriented nature of corporate leadership, where decisions and strategies are essential for navigating competitive markets. Additionally, the emphasis on personal traits underscores the importance of cognitive and emotional capabilities in corporate environments.

Figure 1C shows the thematic distribution from interviews with journalists regarding their understanding of leadership. The “actor” theme (47.95%) remains the most prominent, similar to politicians and corporate leaders, focusing on notable figures such as government officials, company executives, and public personalities. Additionally, “leadership activities” (10.33%) and “achievement” (10.33%) are equally emphasized, indicating that journalists associate leadership with both the actions leaders take and the results they produce. The third notable theme, “context” (10.12%), suggests that journalists are keenly aware of the socio-political environment in which leadership occurs, much like politicians. Journalists’ view of leadership closely mirrors corporate leaders, particularly in their focus on prominent individuals and leadership activities. However, their equal emphasis on achievements highlights the media’s evaluative role in assessing leaders’ success or failure. The inclusion of context as a significant theme reflects journalists’ understanding that leadership is shaped by external factors such as political climates and public opinion. This suggests that media professionals report on leaders and contextualize their actions within broader societal frameworks.

### 4.1 Conceptual framework

In this study, the content analysis method was used to examine the concept of leadership in depth. Content analysis is a method that allows inferences to be made about the message by examining the presence and relationships of certain words and expressions in communication (Nienaber, 2010). The analysis process of the study includes stages such as determining text blocks, defining analysis units, open coding, creating a coding scheme, and detecting patterns. The data obtained from face-to-face interviews were deciphered and analyzed. Words and phrases associated with the research questions were carefully selected and coded (Krippendorff, 2004). During the coding process, the researchers performed the coding process independently and came together after the open coding phase to agree on the coding scheme. Later, the axial coding process was initiated, detailed discussions were carried out on each theme, and a consensus was reached (Graneheim and Lundman, 2004).

#### 4.1.1 Coding categories and comments

The participants highlighted prominent leadership figures, including individuals (e.g., Mustafa Kemal Ataturk, Turgut Ozal), groups (e.g., society, family), and institutions (e.g., TUSIAD). The category of actors reveals how leadership identity is shaped by interactions between individuals, groups, and institutions. The historical and cultural context is critical to understanding the roles of leaders in society.

The basic qualities that leaders should have are knowledge, honesty, courage, and self-discipline. Honesty and courage strengthen leaders’ moral authority, while knowledge and self-discipline are seen as indispensable elements for making strategic decisions. These qualities encompass both the ethical and practical dimensions of leadership identity.

Leaders’ activities include goal-setting, problem-solving, and decision-making. This reveals that leadership requires certain characteristics and is also an action-oriented process. Leadership activities play a vital role in guiding organizations and societies.

The category of success includes evaluating leaders based on concrete outputs. Factors such as creating change, gaining effectiveness, and gaining power are among the success criteria of leaders. These achievements reflect the ability of leaders to initiate societal or institutional transformations by developing innovative approaches.

Leadership is influenced by geographical, political, and societal environments. This study emphasizes the relationship of leadership identity with environmental conditions. For example, factors such as democracy, economic conditions, and social context are among the important factors that shape leadership processes. Leaders need to act in accordance with these factors.

#### 4.1.2 Development of the conceptual framework

In line with these findings, a multidimensional conceptual framework has been developed for leadership identity construction. This framework proposes that leadership be associated with external factors such as individual characteristics (e.g., knowledge, integrity), activities (e.g., decision-making, crisis management), achievements (e.g., change-making, effectiveness), context (e.g., political and economic conditions), and media. In particular, it is emphasized that leadership identity is shaped at the intersection of individual qualities and external environmental factors (Yukl, 2022; Northouse, 2021).

Media influence is one of the important factors in leadership identity. The media is critical in shaping leaders’ public perception and positioning them in a societal context (Fairhurst and Connaughton, 2014). In this context, the media stands out as a tool that strengthens leaders’ communication strategies and the ties they establish with society.

This conceptual framework contributes to the leadership literature as a complex interplay of individual characteristics and societal contexts. The study reveals how leadership goes beyond individuals’ qualities and is shaped by social, political, and cultural contexts. This approach allows for developing a more comprehensive and contextual understanding of leadership work.

## 5 Limitations and prospects

The findings from this study suggest that leadership identity is not static; instead, it is dynamic and shaped by the ongoing interaction between leaders and their environments. Future research should explore how digital media and online platforms further influence leadership identity construction. As leaders increasingly engage with their audiences through digital channels, understanding how these platforms impact public perception and leadership practices is crucial.

Another area for future exploration is the role of cultural differences in leadership identity construction. While this study focuses on Turkish leaders, cross-cultural comparisons could offer deeper insights into how diverse cultural values and societal norms shape leadership.

## 6 Conclusion

The study comprehensively explains leadership identity across different sectors, including politics, business, and journalism. Each industry presents unique perspectives on leadership, shaped by personal traits, professional roles, and societal contexts. By comparing leadership themes derived from interviews with politicians, business leaders, and journalists, this study reveals the commonalities and sector-specific nuances in how leadership is perceived and enacted.

Analyzing themes from interviews with politicians and bureaucrats offers a detailed insight into political leadership identity. Politicians often frame leadership about historical figures and traditional values, which dominate discussions about political leadership. Prominent leaders like Mustafa Kemal Ataturk and Turgut Ozal are frequently referenced, highlighting the symbolic and historical weight attached to leadership in Turkish politics.

Moreover, political leadership is tightly connected to governance, democratic processes, and ethical decision-making. The emphasis on governance, as well as the socio-political environment, suggests that institutional structures, public expectations, and democratic responsibilities shape political leaders. As reflected in the findings of Bilodeau et al. (2019), political leadership in democratic societies hinges on the leader’s ability to navigate institutional mechanisms while maintaining public trust. The emergence of religious themes also points to how political leadership intersects with cultural and ideological contexts, reflecting the broader socio-political landscape of Turkey.

Corporate leadership is notably distinct from political leadership in its focus on personal traits, strategic actions, and adaptability to complex environments. Interviews with company founders, CEOs, and senior executives emphasized personal qualities such as resilience, emotional intelligence, and strategic decision-making. The most significant theme in corporate leadership was the individual leader, underscoring the highly personalized nature of leadership in business sectors.

However, corporate leadership also reflects the broader organizational and stakeholder-driven context in which it operates. Leaders are expected to foster innovation, drive organizational success, and build strong teams. The focus on corporate social responsibility (CSR) initiatives and long-term value creation reflects a growing societal shift toward more socially responsible leadership models. This finding aligns with the study of Carmeli et al. (2013), which highlighted the role of innovation and stakeholder engagement in shaping modern corporate leadership identity.

While business leaders are traditionally evaluated based on profitability, the present study suggests that ethical responsibility, emotional intelligence, and the ability to navigate global competitiveness are increasingly important. These traits and actions illustrate a shift toward more holistic evaluations of leadership, where long-term societal well-being is considered alongside financial success.

Journalists’ perspectives on leadership offer a unique view, as their role in framing leadership through media coverage plays a critical part in shaping public perception. The interviews revealed that journalists focus heavily on leadership qualities such as intelligence, emotional awareness, and decision-making. Leadership activities, including team-building and crisis management, are emphasized, particularly in evaluating leaders’ success and effectiveness in public life.

A significant theme in the journalists’ interviews was their role as intermediaries between leaders and the public. This corresponds with the findings of Fairhurst and Connaughton (2014), who explored how media professionals shape leadership identity by framing leaders’ actions through their coverage. Journalists’ focus on external factors such as political climates and societal contexts further underscores their understanding that leadership cannot be viewed in isolation from the broader environment. The growing emphasis on corporate social responsibility in media discourse suggests that ethical leadership is becoming a more prominent criterion for evaluating leadership success.

In summary, the study highlights the multifaceted nature of leadership identity across different sectors. While personal traits such as emotional intelligence and ethical responsibility are essential across all domains, the specific contexts- political, corporate, or journalistic- significantly influence how leadership is enacted and evaluated. Political leaders focus on governance and public service, business leaders emphasize innovation and long-term value creation, and journalists highlight the importance of public perception and media scrutiny in shaping leadership identity. This sector-specific analysis provides a richer understanding of the complexities surrounding leadership in modern society. It paves the way for further research on how leadership continues to evolve in response to societal, technological, and cultural changes.

## Data Availability

The raw data supporting the conclusions of this article will be made available by the authors, without undue reservation.
